# Bimetallic Steels: A Structured Review of Fabrication Routes, Material Properties, and Component Performance

**DOI:** 10.3390/ma19122505

**Published:** 2026-06-10

**Authors:** Ziheng Ding, Xuanyi Xue, Fei Wang, Neng Wang, Shuai Li, Jianmin Hua

**Affiliations:** 1 School of Civil Engineering, Chongqing University, Chongqing 400045, China; 2 State Key Laboratory of Safety and Resilience of Civil Engineering in Mountain Area, School of Civil Engineering, Chongqing University, Chongqing 400045, China; 3 School of Management Science and Real Estate, Chongqing University, Chongqing 400045, China; 4 Department of Civil Engineering, The University of Hong Kong, Pokfulam Road, Hong Kong, China

**Keywords:** bimetallic steel, fabrication process, mechanical behaviour, durability, structural applications

## Abstract

Bimetallic steel, as a layered composite material formed by metallurgically bonding two dissimilar metals, combines the excellent corrosion resistance of the cladding layer with the superior mechanical properties (such as high strength and toughness) of the base layer. It has been widely applied in demanding fields like marine engineering, the petrochemical industry, and energy equipment, where comprehensive material performance is critical. This paper provides a structured review of the research progress and application status of bimetallic steel. First, mainstream fabrication techniques, such as explosive welding and roll bonding, along with their effects on interfacial bonding quality, are analyzed. Subsequently, key material characteristics, including welding performance, mechanical properties, and corrosion behavior, are discussed. Furthermore, the component-level bearing performance and failure mechanisms under various loading conditions are evaluated. Finally, by synthesizing existing research, current knowledge gaps in areas like long-term service life assessment, adaptability to extreme environments, and efficient intelligent manufacturing are identified, and future development trends are outlined. This review provides important academic reference and engineering guidance for deepening the understanding of bimetallic steels and promoting their safer, more reliable, and cost-effective application in major engineering projects.

## 1. Introduction

**Figure 1:**
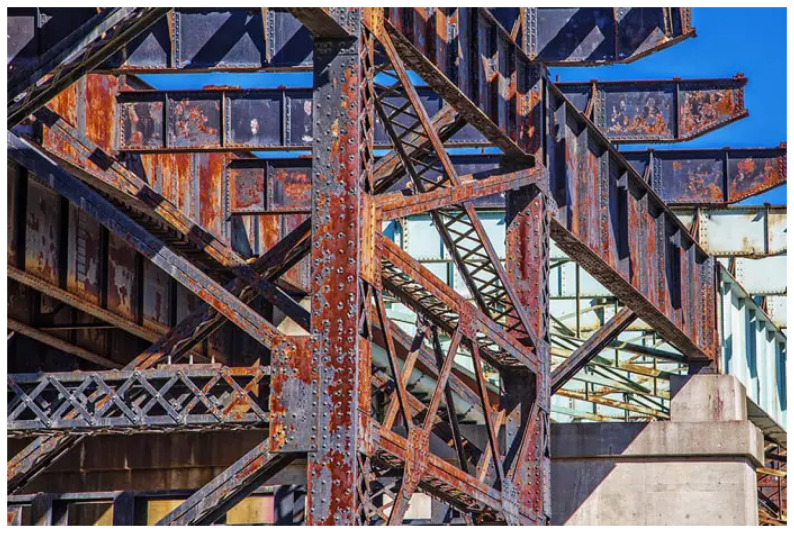
Corrosion damage in conventional steel structures.

Steel structures have been widely used in modern buildings and bridge engineering due to their outstanding advantages, including a high strength-to-weight ratio, construction efficiency, and architectural flexibility. However, conventional carbon steels and low-alloy steels generally exhibit poor corrosion resistance under natural exposure and harsh service environments, and are therefore susceptible to severe corrosion, as shown in Figure 1. Such deterioration can lead to a reduction in the effective cross-sectional area of structural members, a decline in load-bearing capacity, a significant increase in life-cycle maintenance costs, and a shortened service life of the structure [1,2]. To improve the durability of steel structures at the material level, high-performance steels such as stainless steel and weathering steel have been gradually developed and applied in engineering practice. Nevertheless, these materials usually involve high alloying-element contents and complex production processes, resulting in high initial costs and limited economic competitiveness. Therefore, achieving an effective balance between superior performance and cost control has become a key issue in promoting the sustainable development of high-performance steel structures.

**Figure 2:**
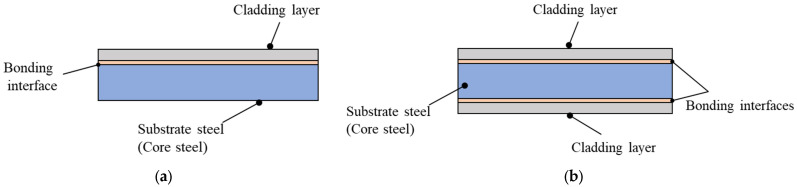
Schematic illustration of the layer configuration and bonding interfaces of clad bimetallic steel plates: (**a**) Single-sided clad steel; (**b**) Double-sided clad steel.

Against this background, layered metallic composites, particularly bimetallic steels, have emerged as a promising solution that combines both performance and economic benefits [3]. The layer configuration and bonding interfaces of clad bimetallic steel plates are shown in Figure 2. This configuration not only preserves the functional properties of the cladding layer, such as corrosion resistance and wear resistance, but also takes advantage of the mechanical performance and cost efficiency of the substrate, thereby producing a pronounced complementary effect in overall performance. In this review, bimetallic steels refer to steel-based layered or graded metallic systems that are produced through fabrication processes such as roll bonding and explosive welding, by which a high-performance cladding material is metallurgically bonded to a conventional structural-steel substrate at the interface [4,5]. Pure coatings, mechanically fastened laminates, and non-steel-based bimetallic systems are not the main focus of this review.

**Figure 3:**
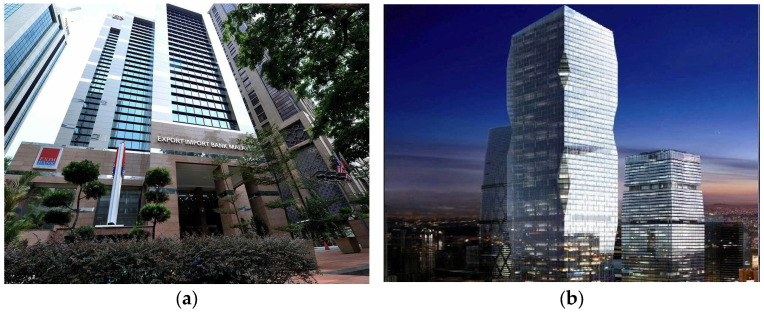
Representative engineering applications of bimetallic steel in building structures: (**a**) Export-Import Bank of Malaysia; (**b**) Guangzhou R&F Yingkai Plaza.

As shown in Figure 3, bimetallic steels have shown increasing application potential owing to their favorable overall performance and economic efficiency in the fields of buildings and infrastructure [6,7]. Their use has extended from exposed structural components such as architectural curtain walls and bridges to more demanding service conditions in marine engineering, petrochemical facilities, pressure vessels, transmission pipelines, and wear-resistant components. In building envelope systems, bimetallic steels can simultaneously satisfy requirements for surface appearance, flatness, and corrosion resistance. In bridge engineering, their superior corrosion resistance can effectively mitigate section loss and performance degradation caused by environmental attack, thereby extending service life and reducing life-cycle cost. In addition, in pipelines, vessels, and marine structures, a rational combination of substrate and cladding materials enables the simultaneous optimization of load-bearing capacity, corrosion resistance, and wear resistance, demonstrating clear engineering applicability [8,9].

**Figure 4:**
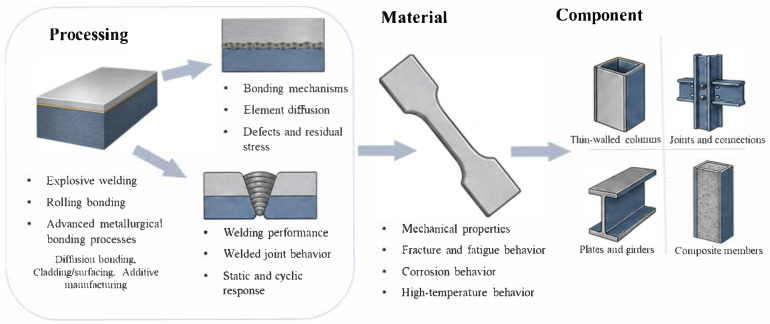
Research framework of bimetallic steels from processing to material and component performance.

Bimetallic steels integrate the structural performance and economic advantages of the substrate with the functional characteristics of the cladding layer, and thus represent an important route toward the coordinated achievement of high performance and cost-effectiveness in steel structures [10,11,12]. However, their service performance is not a simple superposition of the properties of the constituent materials, but rather is governed by the coupled effects of fabrication processes, interfacial microstructure, elemental diffusion, phase transformation, and subsequent processing. Substantial progress has been made in areas such as explosive welding, roll bonding, surfacing-based fabrication, and interfacial characterization. Existing studies are still largely focused on individual aspects, such as process mechanisms, interface analysis, or single-property evaluation [13,14,15]. Previous reviews have provided valuable summaries of clad-steel material properties, corrosion-resistant cladding techniques, metallurgical bonding, and welding-related issues [4,16,17]. However, these reviews mainly focused on fabrication methods, interfacial metallurgy, welding issues, or material-level properties. A broader discussion from both material and component perspectives remains limited. To address this gap, the present review examines bimetallic steels within a process–material–structural performance framework (Figure 4). This framework is used to organize the discussion of fabrication routes, interface characteristics, material behavior, component response, and potential engineering implications. In this way, the review aims to provide a broader perspective for understanding common mechanisms, service limitations, and future research needs in bimetallic steel systems.

This paper is designed as a structured and narrative review. The literature search was conducted using Web of Science, Scopus, ScienceDirect and major publisher databases. The search terms included “bimetallic steel”, “clad steel”, “stainless-clad steel”, “titanium-clad steel”, “explosive welding”, “roll bonding”, “interface”, “mechanical properties”, “fatigue”, “corrosion”, “high temperature”, “buckling”, “plate girder”, “joint”, and “composite member”. The included studies mainly involve steel-based layered or graded metallic systems with metallurgical bonding. Studies on fabrication routes, interface evolution, welding, mechanical properties, fatigue, corrosion, high-temperature behaviour, and structural performance were considered. Pure coatings, mechanically fastened laminates, non-steel-based bimetallic systems, and studies without clear relevance to bimetallic steel materials or structures were excluded. Because the available studies differ in material system, clad ratio, fabrication route, specimen geometry, and loading or exposure condition, this review emphasizes representative trends, mechanisms, and indicators rather than statistical pooling.

## 2. Material Systems and Fabrication Techniques

### 2.1. Explosive Welding

**Figure 5:**
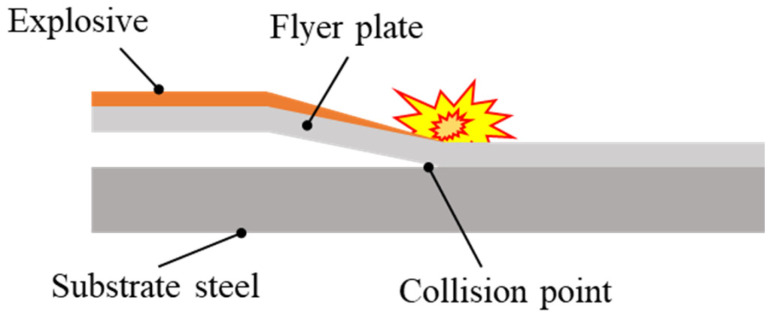
Schematic illustration of the explosive welding process.

Explosive welding is a typical solid-state route for producing steel-based bimetallic composites, especially when conventional fusion welding is restricted by metallurgical incompatibility or brittle intermetallic formation [8,13,18,19]. As shown in Figure 5, the flyer plate is accelerated by detonation and collides obliquely with the base plate at high velocity, generating intense local pressure and jetting at the collision front, thereby forming a metallurgical bond within an extremely short timescale [13,20]. Although the process is generally classified as solid-state joining, local melting and intermixing may still occur in limited interfacial regions under excessive collision energy [18,21,22]. The bonding quality is mainly controlled by explosive ratio, detonation velocity, stand-off distance, flyer thickness, collision angle, and collision-point velocity, which together define the effective welding window [13,18,20]. In practice, the design of explosive welding increasingly relies on combined theoretical analysis, numerical calculation, and experiment, rather than empirical trial-and-error alone [20].

A characteristic feature of explosive cladding is the evolution of the interface from a smooth or straight morphology to a wavy morphology as collision energy increases [8,13,15,21]. The wavy interface is generally preferred because it enlarges the bonding area and promotes mechanical interlocking, which usually improves interfacial strength [8,13]. However, a smooth interface can also provide satisfactory bonding when the collision conditions remain within an appropriate welding range [8]. Another common feature is the formation of a severely deformed interfacial zone. Grains adjacent to the bond line are often elongated or refined, and the hardness usually reaches a maximum near the interface before gradually decreasing toward the plate interior [13,21,23,24]. This interfacial hardening is mainly associated with shock-induced plastic deformation and localized microstructural refinement [21,23].

For stainless-clad bimetallic steels, the main metallurgical concern is usually the control of wave morphology, local hardening, and bonding uniformity [8,21,24]. By contrast, titanium-clad bimetallic steels are more sensitive to local intermixing because vortex zones, islands, or folded regions may contain brittle Fe–Ti intermetallics such as FeTi and Fe_2_Ti under high-energy conditions [13,18,22]. These phases are hard and brittle, and therefore may reduce local toughness even when the overall bond strength remains high [22]. Post-weld treatment is therefore important in many explosively bimetallic steels. Heat treatment can reduce residual stress and interfacial hardening, transform unstable vortex structures, and improve toughness, although some decrease in shear strength may occur simultaneously [23,24]. In addition, explosive welding is often combined with subsequent rolling or shaping, indicating that it should be viewed not only as a joining method, but also as a preparatory route for further thermomechanical processing of clad products [25,26,27]. Overall, the essential issue in explosive cladding is the balance among process window, interfacial morphology, and local metallurgical reaction [18,20,22]. For steel-based clad systems, process optimization should therefore focus on controlling collision energy to ensure sufficient bonding while suppressing excessive melting, brittle intermetallic formation, and over-hardening at the interface [13,22,24].

### 2.2. Roll Bonding

**Figure 6:**
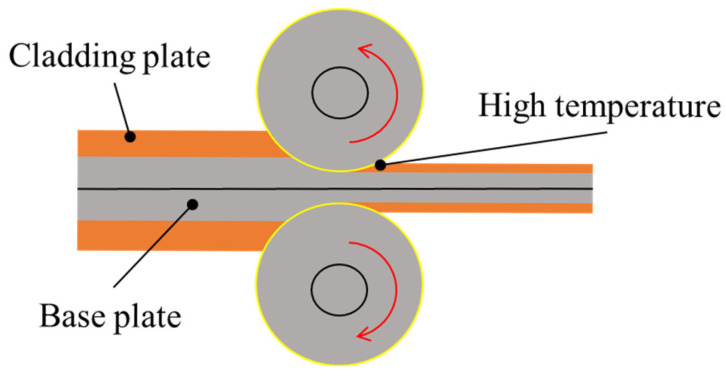
Schematic illustration of the hot roll bonding process for bimetallic steel plates.

Roll bonding is one of the most practical routes for manufacturing bimetallic steels because it enables solid-state joining with relatively high efficiency, low environmental burden, and good scalability for industrial production [5,14,28]. Compared with explosive bonding, it is more suitable for continuous processing and dimensional control, whereas compared with diffusion bonding, it is more attractive for large-scale products [5]. The hot roll bonding process for bimetallic steel plates is shown in Figure 6. As a result, roll bonding has become a major research focus for both stainless steel and titanium-clad bimetallic steels [5,28,29]. The quality of roll-bonded interfaces is governed by the combined effects of surface cleanliness, atmosphere control, rolling temperature, and reduction ratio [28]. In essence, bonding is achieved through oxide-film disruption, exposure of fresh metal, intimate interfacial contact, and diffusion-assisted metallurgical joining under high-temperature compressive deformation [28,30]. Accordingly, surface preparation and vacuum sealing are indispensable, especially for Ti/steel combinations, where oxidation during heating readily deteriorates bonding [31].

A central feature of roll bonding is that improved diffusion does not always lead to better performance. In stainless-steel clad plates, appropriate rolling conditions promote Fe, Cr, and Ni interdiffusion and thus strengthen the interface, but carbon migration simultaneously produces a decarburized layer on the carbon steel side and a carburized layer on the stainless steel side [28,32]. These transition layers are intrinsic to the process and strongly influence local hardness, toughness, and corrosion behavior. Therefore, the key issue is not to maximize diffusion, but to obtain sufficient metallurgical bonding while limiting harmful carbon redistribution. This balance is even more critical in Ti/steel systems. Moderate temperatures and sufficient reduction can improve bonding by enhancing interfacial contact and diffusion, whereas excessive temperatures promote the formation of brittle reaction products such as TiC, FeTi, and Fe_2_Ti, which markedly reduce bond strength [30,31,33]. Hence, the processing window for Ti/steel roll bonding is much narrower than that for stainless/carbon steel systems, and the main challenge is to suppress interfacial embrittlement while retaining adequate joining strength.

Moreover, interlayer design is often adopted for this purpose. Interlayers such as IF steel, V, and Ni can modify diffusion paths and interfacial phase evolution, thereby improving bond quality under suitable conditions [31,33]. However, their effect is not unconditional, since new reaction products may still form when the thermal exposure becomes excessive. Thus, interlayers should be viewed as a means of regulating the bonding window rather than a complete solution to interfacial reactions. Current studies indicate that roll bonding is a robust and versatile fabrication route, but its success depends on precise control of interfacial reactions and deformation compatibility. For stainless-clad bimetallic steels, emphasis should be placed on controlling carbon-induced transition layers and carbide precipitation. For titanium-clad bimetallic steels, the priority is to limit brittle Ti–Fe and Ti–C compounds. Future development is expected to focus on tighter process control and on extending roll-bonding concepts from plates to section steels, rods, shafts, and tubes [34,35,36].

### 2.3. Advanced Metallurgical Bonding Processes

**Figure 7:**
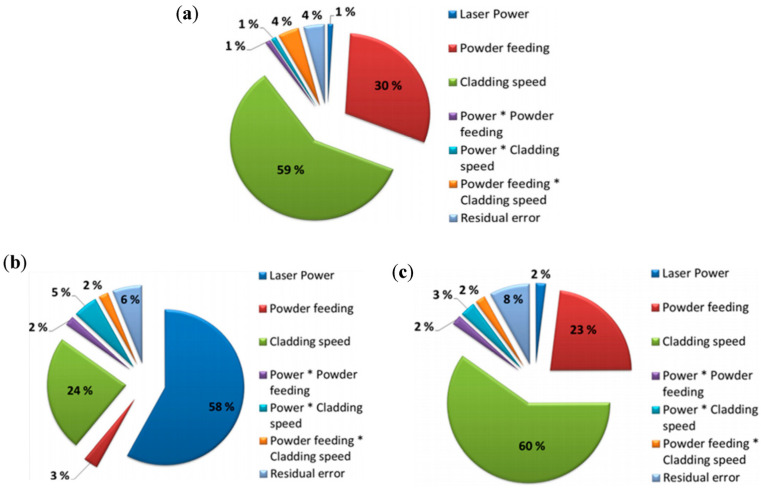
Effects of laser cladding process parameters on coating geometry: (**a**) coating height; (**b**) coating width; and (**c**) wetting angle between the coating and substrate. The asterisk (*) denotes the interaction between two process parameters. Adapted from Han et al. [41].

Beyond explosive welding and roll bonding, emerging fabrication routes have further expanded the processing window of bimetallic steels, especially for thick sections, tubular products, large components, and complex geometries. On the one hand, advanced metallurgical bonding routes, including electroslag-assisted processing, surfacing plus hot rolling, and casting-derived liquid–solid composite methods, rely on controlled local melting, interfacial diffusion, and subsequent thermal or thermomechanical regulation to obtain sound bonding and graded transition zones [37,38,39,40]. Related laser-assisted cladding of steels has also been widely investigated, and its coating geometry and dilution behavior are highly sensitive to process parameters, as summarized in Figure 7 [41]. On the other hand, additive manufacturing provides a flexible route for producing bimetallic steels with locally tailored properties and complex shapes. In these systems, interfacial integrity is mainly governed by melt pool behavior, elemental diffusion, thermal cycling, and post-build heat treatment. Studies on laser powder bed fusion of T91/316H and 316L/1.2709 showed that process parameters strongly affected defect formation, interfacial mixing, and mechanical response, while post-build heat treatment further modified the strength–ductility balance [42,43,44]. For larger structures, arc-based routes such as CMT-WAAM (Cold Metal Transfer Wire Arc Additive Manufacturing) and GMAW (Gas Metal Arc Welding) produced defect-free or nearly defect-free interfaces in 316LSi/ER70S-G and 316L/LCS bimetals, with diffusion-induced interfacial hardening and favorable tensile performance [45,46]. Hybrid additive routes were also reported, as forged SS304L combined with WAAM-deposited SS308L showed that pulse-mode deposition could further refine the interfacial structure and improve mechanical properties [47]. These emerging routes extend bimetallic steel fabrication beyond conventional explosive welding and roll bonding. However, their reliability still depends on interfacial diffusion, phase evolution, residual stress, and post-process regulation. For additive manufacturing, repeated thermal cycling, dilution, defects, cracking, anisotropy, and scalability remain key issues. Therefore, in structural bimetallic steel applications, additive manufacturing should be regarded as a promising but still developing supplement to explosive welding and roll bonding.

### 2.4. Welding Performance

The welding of bimetallic steel plates requires careful control of pass sequence and filler metal selection, because dilution between the backing steel, cladding layer and deposited weld metals can strongly affect the fusion zone, heat-affected zone and corrosion resistance [17]. The welding of stainless-clad steel plates is mainly challenged by the compositional and thermo-physical mismatch between the carbon-steel substrate and the stainless-steel cladding [16]. Existing studies indicate that filler metal selection and welding sequence are critical [48,49,50]. From a structural engineering perspective, more studies have shifted from weld qualification to the combined assessment of load-bearing capacity, welding configuration, and fabrication efficiency [51,52].

**Figure 8:**
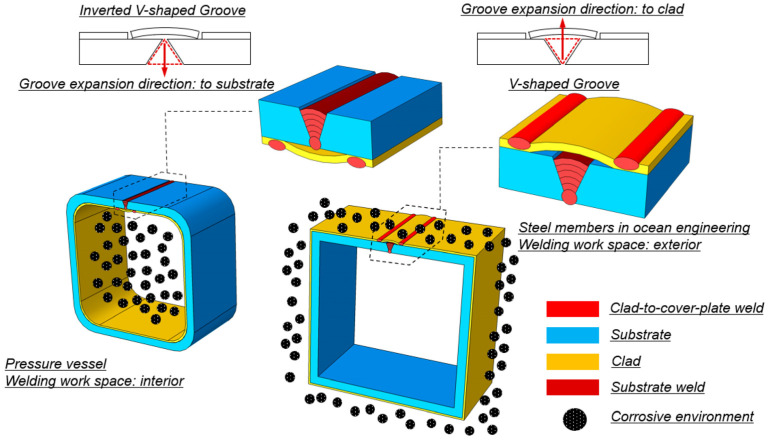
Groove configuration and welding arrangement of titanium-clad bimetallic steel joints for ocean engineering applications. Adapted from Jiang et al. [58].

For titanium-clad bimetallic steel plates, welding is mainly constrained by the poor metallurgical compatibility between Ti and Fe [53]. Direct Ti–Fe mixing promotes brittle intermetallic compounds, which reduce ductility and induce cracking. Therefore, current studies mainly focus on filler design and transition-layer control [54,55,56,57]. Structural joint tests showed that Type II and Type III TA2/Q355B butt-welded joints (Figure 8) could reach about 90% and 93% of the parent-metal strength, respectively, but with clearly reduced elongation [58]. Multi-principal or high-entropy fillers can further improve joint strength by regulating weld phases and interrupting brittle-phase continuity [59]. However, high-hardness zones and crack sensitivity may still remain near the TA2-side transition zone, indicating that local embrittlement has not yet been fully eliminated [60].

Building on static joint studies, further study has examined the cyclic and fatigue degradation of bimetallic steel welds [61]. Ban et al. [62] showed that butt-welded 316L/Q235B stainless-clad steel joints exhibited good cyclic integrity and energy dissipation, with no weld fracture or interfacial delamination under cyclic loading. Yang et al. [63] further found that the low-cycle fatigue life of S31603/Q355B welded joints was mainly controlled by welding configuration, dissimilar-metal fusion, local hardening, and crack propagation. For titanium-clad bimetallic steel plates, Hai et al. [64] established S–N curves for TA2/Q355B butt-welded joints and showed that fatigue cracking was dominated by the titanium cap-to-clad weld rather than interfacial delamination. In bridge applications, Liao et al. [65,66] investigated stainless-clad bimetallic steel rib-to-deck welded joints and reported good fatigue resistance; meanwhile, the stainless-steel cladding layer reduced peak tensile residual stresses near the weld.

From a fabrication perspective, these studies in Section 2 show that processing routes should not be evaluated only by bonding strength. Their effects on interface morphology, residual stress, interfacial phases, and subsequent structural performance should also be considered. Explosive welding is suitable for highly dissimilar metals because it can form strong metallurgical bonding within a short time. However, excessive collision energy may cause local melting, unstable wavy interfaces, high residual stresses, and brittle intermetallic compounds. Roll bonding is more suitable for large-scale plate production and dimensional control, but its bonding quality is sensitive to surface preparation, reduction ratio, rolling temperature, and carbon diffusion. Advanced bonding and additive manufacturing routes provide greater flexibility for thick sections, tubular products, and graded components. However, their reliability is still affected by thermal cycling, dilution, residual stress, and process repeatability. Therefore, the preferred fabrication route depends on the target service condition. Corrosion-resistant plates require continuous and defect-free cladding layers. Fatigue-sensitive members require limited interface defects and controlled residual stresses. High temperature or welded components require stable interfacial phases and restricted brittle reaction zones.

## 3. Material Properties

### 3.1. Mechanical Properties

The overall tensile response of bimetallic steel is commonly estimated using a mixture-based rule, in which the stresses of the carbon steel substrate (*σ_s_*) and cladding (*σ_c_*) are weighted by the clad ratio *β*, as expressed in Equation (1). For the cladding metal, which usually exhibits continuous yielding, such as stainless steel, the 0.2% proof strain *ε_c_*_,0.2_ can be determined from Equation (2), where the elastic strain corresponding to the 0.2% proof stress *σ_c_*_,0.2_ is combined with a plastic offset of 0.002. *E_c_* denotes the elastic modulus of the clad section. Stainless-clad bimetallic steel generally exhibits mechanical properties between those of the stainless steel cladding and carbon steel substrate, and its response is mainly governed by the clad ratio and interfacial condition [67].

(1) $$\sigma_{sc}=\sigma_{s}(1-\beta )+\sigma_{c}\beta$$

(2) $$\varepsilon_{c,0.2}=\frac{\sigma_{c,0.2}}{E_{c}}+0.002$$

With increasing clad ratio, the stress–strain curve gradually changes from the yield-plateau feature of carbon steel to the continuous hardening behavior, while the elastic modulus usually decreases and the equivalent yield strength increases [68,69]. For example, in stainless-clad plates tested by Tang et al. [68], increasing the clad ratio from 0.21 to 0.55 increased the yield strength from 315.9 MPa to 359.5 MPa and the ultimate strength from 503.6 MPa to 621.1 MPa, whereas the elastic modulus decreased slightly from 205.1 GPa to 199.6 GPa. Existing studies further showed that mixture-based methods can reasonably describe the overall tensile response, although element diffusion during bonding may produce interfacial transition zones and local property gradients, such as decarburized and carburized layers [70,71]. In addition, improved metallurgical bonding can enhance deformation compatibility and tensile ductility by suppressing interfacial delamination and premature localized necking, whereas excessive thermal exposure may reduce strength because of recovery and grain coarsening near the interface [72]. Recent studies also indicated that stainless-clad bimetallic steel is strain-rate sensitive, but the overall trend remains similar, namely an intermediate constitutive response between the two constituent metals, with clad ratio still being an important controlling parameter [11,73,74,75]. Accordingly, constitutive models have mainly focused on clad-ratio-dependent prediction of the full-range stress–strain response under both static and dynamic loading [73,74,76]. A summary of the material combinations, fabrication processes and loading conditions reported in existing studies is provided in Table 1.

### 3.2. Fracture and Fatigue Behavior

The bimetallic steels exhibit distinct cyclic responses from monolithic steels. The cyclic behaviour is governed by the clad ratio, constitutive hardening characteristics, and crack evolution. Earlier fracture mechanics studies on layered steel composites and bimaterial plates showed that fatigue crack propagation is affected by residual stress, plastic mismatch, crack-growth direction, and interface constraint [77,78,79]. The experimental conditions of representative studies on fracture and fatigue behavior are summarized in Table 2. Existing results indicate clear hysteretic behaviour, cyclic hardening/softening, and good energy dissipation capacity [80,81,82]. The clad layer can improve ductility and delay crack propagation, leading to enhanced low-cycle fatigue resistance. The Ramberg–Osgood and Chaboche-type models have been shown to provide reasonable predictions of the cyclic response and constitutive evolution of these materials. Liao et al. [83] further showed that the low-cycle fatigue behaviour of hot-rolled 304 + Q235B stainless-clad steel was still dominated by the carbon-steel substrate. Cracks mainly initiated from the Q235B side, while no obvious interfacial debonding was observed, indicating stable composite action under cyclic loading. Kang et al. [84,85] extended the study of laser-cladding-produced stainless-clad bimetallic steels by showing that these materials exhibit stable and full hysteresis loops, with increasing clad ratio leading to a stronger contribution of kinematic hardening to the overall response.

**Figure 9:**
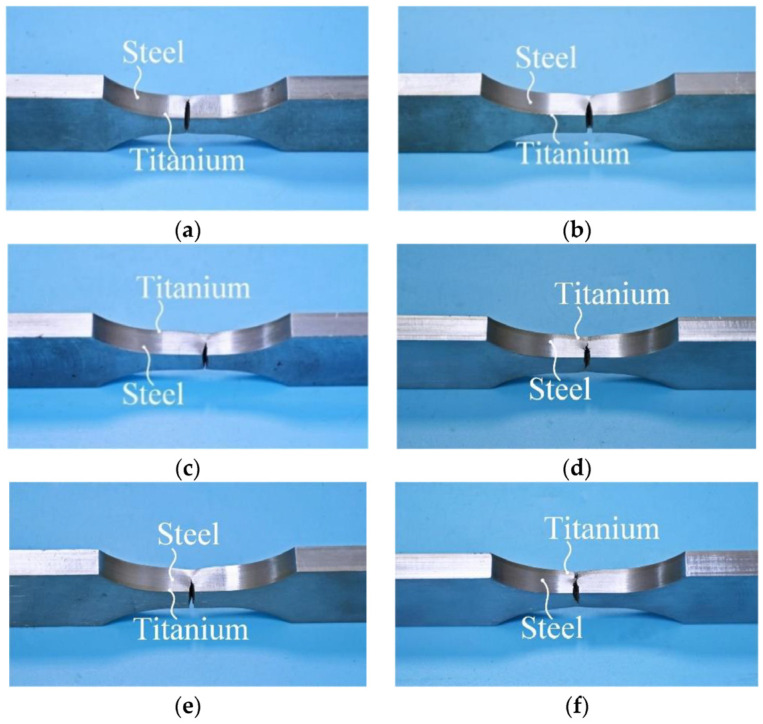
Representative fatigue failure modes of titanium-clad bimetallic steels with different interfacial conditions: (**a**) explosion-bonded specimen; (**b**,**c**) hot-rolled specimens with low bonding strength; and (**d**–**f**) hot-rolled specimens with high bonding strength. Adapted from Jiang et al. [91].

Hai et al. [86,87,88] systematically investigated hot-rolled titanium-clad bimetallic steel from cyclic constitutive response to low-cycle fatigue and large-strain reversals. Their studies showed stable and relatively full hysteresis loops, evident path-dependent cyclic behaviour, and generally limited isotropic hardening, with pure or dominant kinematic hardening being sufficient for modelling in many cases. High-cycle fatigue studies on titanium-clad bimetallic steel indicate that its fatigue behaviour is strongly controlled by the bonding interface, manufacturing route, clad ratio, and surface condition [89,90,91]. The typical fatigue failure modes of titanium-clad bimetallic steels with different interfacial conditions are shown in Figure 9. Fatigue cracks mainly initiate at the bonding interface or the steel-side surface, while higher interfacial bonding strength and surface treatment can improve fatigue resistance, whereas increasing the clad ratio generally reduces fatigue strength. A further study on HRB400E/316L stainless steel clad rebar showed that strain amplitude remained the dominant parameter, whereas strain rate and bar diameter also influenced fatigue life and degradation behaviour [92].

**Table 2:** Summary of representative studies on the fatigue and fracture behaviour of bimetallic steels.

References	Bimetallic Steel Grade	Fabrication Route	Loading Condition
Liu et al. [82]	Stainless-clad bimetallic steels	Metallurgical bonding	Cyclic behaviour with various clad ratios
Ban et al. [81]	Q235B + 316L	Hot-rolled bonded	Low-cycle fatigue under constant strain amplitudes
Ban et al. [80]			Monotonic tension/compression and cyclic loading test
Liao et al. [83]	Q235B + 304		Low-cycle fatigue under different strain ratios
Kang et al. [84]	Q355 + 316L	Laser cladding	Cyclic loading under seven strain-controlled protocols
Kang et al. [85]			Metallographic characterization, and strain-controlled uniaxial cyclic tests
Hai et al. [86]	Q345B + TA2	Hot-rolled bonded	Cyclic constitutive behaviour under cyclic loading
Hai et al. [88]	Q355B + TA2	Hot-rolled bonded	Cyclic behaviour under large plastic strain
Hai et al. [87]			Low-cycle fatigue with different strain amplitude
Jiang et al. [91]			High-cycle fatigue with different interfacial conditions
Ban et al. [90]			High-cycle fatigue with different clad ratios
Huang et al. [89]		Explosive bonded	High-cycle fatigue; axial force-controlled test
Li et al. [92]	HRB400E + 316L	Hot-rolled bonded	Low-cycle fatigue with buckling

### 3.3. Corrosion Behavior

Regarding the corrosion resistance of bimetallic steel plates, existing studies have mainly focused on the corrosion response of stainless-clad steel plates in chloride-containing or simulated marine environments (Table 3). The available results consistently show that the stainless steel cladding can markedly improve the overall corrosion resistance of the material, whereas the galvanic effect at the bimetallic interface is a key factor governing its long-term durability [93,94]. However, this improvement is also affected by the surface state and metallurgical changes induced by the cladding process. For example, electrochemical tests on explosion-clad carbon steel/316L stainless steel plates showed that the cladded 316L layer exhibited lower corrosion resistance than non-cladded 316L steel in sulphuric acid solution, indicating that deformation, surface defects, and passive-film stability should also be considered [95]. Meanwhile, the cladding ratio affects not only the initial hardness and mechanical properties, but also the retention of these properties after corrosion. When the interface is exposed, the enlarged cathodic area may also intensify local galvanic corrosion [94]. In addition, studies on pre-corroded specimens have shown that corrosion pits can promote crack initiation and significantly shorten low-cycle fatigue life, indicating that corrosion damage can further evolve into service performance degradation [96].

**Table 3:** Summary of representative studies on the corrosion behaviour of bimetallic steels.

References	Bimetallic Steel Grade	Fabrication Route	Corrosion Type/Environment	Main Topic
Zhang et al. [93]	Q235 + 304	Hot-rolled bonded	Neutral salt spray	Mainly mass loss, electrochemical tests, Microstructural and phase analyses.
Zhu et al. [94]	Q345 + 316L	Hot-rolled bonded		Tensile tests after different exposure durations.
Chen et al. [96]	Q420qENH + 316L	Hot-rolled bonded	Dry–wet alternating pre-corrosion in NaHSO_3_ solution	Tensile tests and strain-controlled low-cycle fatigue tests
Sawicki [97]	C45 + 304L (Steel bar)	Explosive bonded + Hot rolling	Acidified salt spray	Corrosion resistance and microstructure
Hua et al. [1,2]	HRB400 + S30408 (Steel bar)	Hot-rolled bonded	Accelerated electrochemical corrosion in NaCl	Tensile tests after corrosion/Finite-element analysis
Wang et al. [98]	HRB400 + S30408 (Steel bar)	Hot-rolled bonded		
Zhou and Ding [99]	45 carbon steel + 304 (Bolts)	Hot-rolled bonded		Tensile test and rotary bending fatigue
Xia et al. [100]	C110 + stainless steel (Tube)	Electroslag remelting + Hot-rolled bonded	Electrochemical corrosion evaluation of stainless-steel inner surface	Corrosion–mechanical property coupling

**Figure 10:**
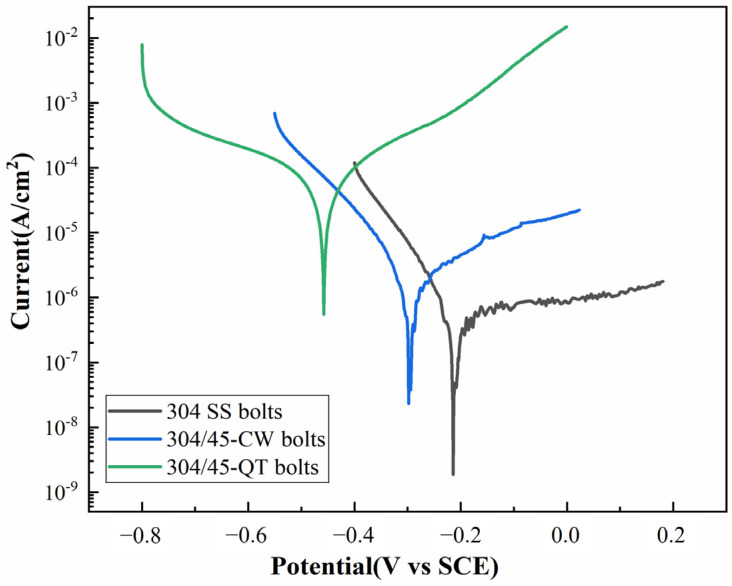
Polarization curves of stainless steel and stainless-clad composite bolts in 3.5 wt.% NaCl solution. Adapted from Zhou and Ding [99].

Research on the corrosion behaviour of stainless-clad bimetallic reinforcing bars has progressed from basic corrosion-resistance verification to corrosion-induced mechanical degradation and corrosion–fatigue interaction. Existing studies generally show that the stainless steel cladding can markedly enhance the overall corrosion resistance of the bar, provided that a sound metallurgical bond and proper fabrication quality are achieved [97]. With increasing corrosion degree, the yield strength, ultimate strength, ultimate strain, and ductility of the bar all decrease [1]. More studies further suggest that, under realistic service conditions, non-uniform corrosion is more likely to develop, and local damage to the cladding may accelerate deterioration of the exposed carbon steel substrate through galvanic interaction, thereby aggravating mechanical degradation [2,98]. For other product forms, such as clad tubes and composite fasteners, the stainless steel cladding can still provide effective protection under appropriate processing conditions (Figure 10) [99,100].

### 3.4. High-Temperature Performance

High-temperature exposure can significantly alter the strength, stiffness and ductility of steels, which directly affects the fire resistance and post-fire safety assessment of building structures [101,102,103,104]. Therefore, a systematic summary of existing studies on both elevated-temperature and post-fire performance is necessary, as presented in Table 4. Shi et al. have conducted a relatively systematic series of studies on the high-temperature and post-fire performance of bimetallic steels. The residual mechanical properties and fracture mode evolution of hot-rolled titanium-clad bimetallic steel under different exposure temperatures and cooling methods were clarified [105]. Subsequent research then shifted to the interfacial behavior of explosively welded stainless-clad bimetallic steel, elucidating the microstructural evolution in the heat-affected zone and its influence on the post-fire interfacial shear resistance [106]. On this basis, further comparison of the post-fire stress–strain responses and failure modes of explosively welded and hot-rolled stainless-clad bimetallic steels showed that the manufacturing process is a key factor governing interlayer interaction and delamination behavior after high-temperature exposure [107]. The research scope was later extended to cold-formed bimetallic steels, showing that cold forming markedly increases strength, reduces ductility, and makes the fracture mechanism more complex [108,109]. In parallel, Ban et al. [110,111] established an important foundation for this field by systematically investigating the post-fire behavior of hot-rolled stainless-clad bimetallic steels. Xu et al. [112] showed that the post-fire mechanical properties of double-sided 316L-clad bimetallic steels remain largely unchanged below about 700 °C, whereas the residual strengths become increasingly dependent on cooling path at higher temperatures. Dai et al. [113,114] further extended post-fire research on stainless-clad bimetallic steels from base-metal coupons to welded joints and cyclic performance. These studies have advanced the field from macroscopic phenomenological description toward interfacial mechanism-based interpretation and parametric model development.

**Table 4:** Summary of representative studies on the high-temperature performance of bimetallic steels.

References	Bimetallic Steel Grade	Fabrication Route	Cooling Condition	Temperature Range	Loading Condition
Shi et al. [105]	Q235 + TA1	Hot roll-bonded	Air cooling and water cooling	300–900 °C	Monotonic tensile test after cooling
Shi et al. [109]					Monotonic tensile test on corner coupons after cooling
Shi et al. [107]	Q235B + 316L	Explosive bonded			Monotonic tensile test after cooling
Shi et al. [108]		Explosive bonded + cold forming			Monotonic tensile test on corner coupons after cooling
Shi et al. [106]		Explosive bonded		300–1000 °C	Interfacial shear test after cooling
Ban et al. [110]		Hot roll-bonded		100–1000 °C	Monotonic tensile test after cooling
Ban et al. [111]	Q355 + 316L	Hot-rolled bonded		300–1000 °C	Monotonic tensile test after cooling
Xu et al. [112]	Q235/Q345 + 316L				Monotonic tensile test after cooling; some specimens with sustained in-fire tensile stress
Dai et al. [113]	Q355 + 316L				Monotonic tensile test of butt-welded coupons after cooling
Dai et al. [114]					Strain-controlled low-cycle fatigue test after cooling
Ban et al. [115]	Q235B + 316L		At elevated temperatures	100–950 °C	Steady-state monotonic tensile test
Li et al. [116]	Q235 + 304L			20–900 °C	Steady-state monotonic tensile test
Gao et al. [117]	Q235 + 304L			Tensile: 300–800 °C; Interface: 100–800 °C	Steady-state tensile test; interfacial shear test; interfacial bonding test
Tian et al. [118]	Q235B + 304L			20–600 °C	Quasi-static and dynamic compression tests; strain rates of 0.001–3000 s^−1^

Existing studies on the in-fire performance of bimetallic steels show a clear development from elevated-temperature tensile properties of single-sided stainless-clad steels to double-sided systems, clad-ratio effects, and interfacial degradation. They have further extended to fire-path effects, temperature–strain-rate coupling, and cold-formed or dissimilar bimetallic steel systems. More specifically, the degradation laws of strength, stiffness, and constitutive response of stainless-clad bimetallic steels during the heating phase [115,116]. Interfacial shear and bonding behaviours at elevated temperatures have also been examined [117]. In addition, related studies have considered the coupled effects of elevated temperature and high strain rate [118].

Taken together, the reviewed material-level studies in Section 3 show that the performance of bimetallic steels is governed by different mechanisms under different service conditions. Tensile behavior is mainly affected by clad ratio, composite-section effect, and deformation compatibility between the substrate and cladding layer. Fatigue and fracture behavior are more sensitive to interface defects, surface damage, local hardening, and residual stresses, because these factors control crack initiation and propagation. Corrosion resistance depends primarily on the continuity of the cladding layer and the exposure condition of the interface. Once the substrate or interface is exposed, galvanic interaction may accelerate local degradation. High-temperature performance is further governed by phase transformation, elemental diffusion, recovery, grain coarsening, and degradation of interfacial bonding. These findings indicate that bimetallic steels should not be assessed only by nominal strength. A reliable material assessment should consider clad ratio, interface quality, fabrication route, and service environment together.

## 4. Component-Level Performance

### 4.1. General

In this section, previous investigations on bimetallic steel structural components, including axially loaded members, plate elements and girders, joints and connections, and concrete-filled composite members, are summarized and discussed. Existing studies have shown that the structural performance of bimetallic steel members is mainly governed by local buckling, overall buckling, patch loading resistance, joint strength and stiffness, as well as the influence of residual stresses, welding details and material heterogeneity. These findings provide the basis for understanding the structural behaviour of bimetallic steel components and for developing appropriate design approaches.

### 4.2. Thin-Walled Column Members

As shown in Table 5, recent studies on clad bimetallic steel stub columns under axial compression can be more clearly grouped by material system and section form. For stainless-clad bimetallic steel, the available evidence covers built-up SHS (Square hollow section) [119], cold-formed SHS [120,121], double-sided angle sections [6], and welded CHS (Circular hollow section) [122]. For titanium-clad bimetallic steel, current studies have focused on cold-formed angle and channel sections [123] and welded CHS [124]. Across these section types, the compressive response is consistently governed by local buckling, while the ultimate resistance is affected by the combined influence of fabrication-induced material variation, initial geometric imperfections, residual stresses, and clad ratio. More specifically, the role of residual stresses has been examined explicitly for cold-formed stainless-clad bimetallic steel SHS and angle sections. Their influence depends on the fabrication route and stress type, but is generally secondary to section slenderness and material enhancement from forming [6,120,121]. Correspondingly, further research has increasingly proposed cross-section design refinements, including modified effective width methods, revised slenderness limits, and DSM (Direct strength method) for clad bimetallic members [6,119,120,121,124]. By contrast, research on slender clad bimetallic steel columns has so far remained much more limited and has mainly focused on overall buckling rather than cross-sectional local instability. Existing studies on stainless-clad bimetallic steel have examined welded box-section and circular-section columns under axial compression [125,126]. Their global flexural buckling resistance is affected by clad ratio, initial imperfections, residual stresses, and section stiffness, indicating that current carbon-steel or stainless-steel column curves may require further refinement for clad members. Recent studies have shown that stainless-clad welded and cold-formed sections have residual stress distributions different from those of conventional single-steel sections [127,128,129]. This difference is mainly caused by welding, cold forming, and material mismatch between the cladding and substrate layers. International studies on explosion-clad corrosion-resistant alloy and carbon steel plates also showed that tensile residual stresses can be introduced into the clad metals because of severe plastic deformation, thermal gradients, and thermal expansion mismatch [130,131]. These findings provide an important basis for subsequent buckling analysis and numerical modelling.

**Table 5:** Summary of representative studies on thin-walled column members using bimetallic steels.

Research Type	Cross-Section	References	Bimetallic Steel Grade	Loading Condition
Stub column	SHS	Ban and Mei [119]	Q370/Q235 + S31603	Axial compression; local buckling behavior
		Liu et al. [120]	Q235 + 304	
		Zeng et al. [121]	Q235B + S30408	
	CHS	Ban et al. [122]	Q235B + S30408; Q235B + 310S; Q355B + S31603	
		Huang et al. [124]	Q355 + TA2	
	Angle	Liu et al. [6]	Q355 + 304	
	Angle/Channel	Shi et al. [123]	Q235 + TA1	
	SHS	Li et al. [132]	Q235B + 304	Axial compression after chloride-ion salt-spray corrosion
		Li et al. [9]	Q235 + 304	Axial compression after neutral salt spray corrosion
	Angle	Shi et al. [133]	Q235 + TA1	Post-fire concentric axial compression after air cooling
	SHS	Wu et al. [134]	Q235 + 304	Axial compression under fire; restrained column fire test
Slender column	SHS	Ban et al. [125]	Q370qE + S31603	Axial compression; overall flexural buckling
	CHS	Ban et al. [126]	Q235B + S31008	
Thin-walled section	SHS	Zhao et al. [127]	Q370 + 316L; Q235 + 316L	Residual stress measurement by sectioning method
	CHS	Ban et al. [128]	Q235B + S31008; Q355B + S31603	
	SHS	Wei et al. [129]	Q235B + S30408	

Moreover, axial compression studies have also been extended to extreme conditions, especially chloride-induced corrosion and fire exposure. Under corrosive environments, stainless-clad bimetallic square tubes generally show substantially lower corrosion depth and smaller losses in axial strength and ductility than carbon steel counterparts [9,132]. However, deterioration becomes more severe once corrosion penetrates the cladding layer and reaches the substrate. Under fire or post-fire conditions, existing studies indicate that elevated temperature not only changes material response and residual resistance, but may also alter cross-section classification, failure development, and the applicability of existing design methods [133,134].

### 4.3. Plate Elements and Girders

Plate elements are fundamental structural components, whose stability and resistance govern the practical application of bimetallic steels in engineering structures. This issue is closely related to layered-plate mechanics, where layer arrangement and thickness ratio have been shown to influence bending response [135]. Table 6 summarizes the representative studies on bimetallic steel plates and girders, including plate buckling, patch loading, web openings, and shear behaviour. Regarding plate elements, Mei et al. [136] conducted a series of studies on the buckling behaviour of stainless-clad bimetallic steel plates. Their work first established the elastic buckling solutions for simply supported plates and clarified the effects of clad ratio, elastic modulus ratio, and plate slenderness, showing that the buckling stress is governed mainly by the bending stiffness. The study was then extended to outstand plates, for which lower buckling resistance was obtained because of the less favourable boundary condition, although the main parametric trends remained similar [137]. More recently, Ban et al. [138] further investigated the nonlinear buckling of imperfect plates and proposed design formulae capable of accounting for plate slenderness, clad ratio, and imperfection effects.

Regarding plate girders, existing studies have mainly focused on the use of clad bimetallic steel as the web material to improve durability while maintaining adequate structural resistance. For titanium-clad bimetallic steel plate girders, some studies showed that the patch-loading behaviour is broadly comparable to that of conventional steel plate girders, with stable post-buckling resistance and no evident interfacial delamination [139,140]. The presence of web openings requires further attention because openings act as geometric discontinuities and may cause local stress concentration near their corners. For homogeneous isotropic steel plates with square openings, Radojković et al. [141] showed that the stress distribution was strongly affected by the opening position and corner radius. The same research line was further extended to girders with web openings, in which the opening location was found to markedly affect the local instability region and the development of the tension field under patch loading [142]. For stainless-clad bimetallic steel plate girders, some studies showed that both patch-loading and shear resistance decrease approximately linearly with increasing clad ratio when the girder geometry is fixed. Existing code provisions were found to be unsuitable for direct application, and modified design expressions were therefore proposed [143,144].

**Table 6:** Summary of representative studies on plate elements using bimetallic steels.

References	Bimetallic Steel Grade	Element Type	Research Method	Loading Condition
Mei et al. [136]	Stainless-clad bimetallic steel	Simply supported plate	Theoretical analysis + Numerical validation	Uniaxial compression
Mei and Ban [137]	Stainless-clad bimetallic steel	Outstand plate		
Ban et al. [138]	Stainless-clad bimetallic steel	Imperfect plate		
Luo et al. [139]	Q235 + TA1	Plate girder	Test + Numerical analysis	Patch loading
Luo et al. [140]				
Luo et al. [142]		Plate girder with web opening		
Xue et al. [143]	Q690 + S316	Plate girder	Numerical analysis + design method	Patch loading
Hua et al. [144]	Q690 + S31608	Plate girder		Shear loading

### 4.4. Joints and Connections

For bimetallic steel structures, existing studies have also extended to various connections and tubular joints, as summarized in Table 7. Yang et al. [7] examined welded box-section beam-columns and showed that these members possessed good seismic performance, with ductility and energy dissipation governed mainly by the width-to-thickness ratio and axial load ratio. Gao et al. [10] investigated gusset plate to stainless clad CHS-X connections and found that their failure modes and normalized strengths were broadly comparable to those of conventional carbon-steel tubular joints. Guo et al. [145] studied double-sided stainless-clad steel bolted connections and showed that net-section tensile fracture governed the response without interface debonding, while current design rules gave reasonable strength predictions.

**Table 7:** Summary of representative studies on joints and connections using bimetallic steels.

References	Bimetallic Steel Grade	Element Type	Research Method	Loading Condition
Yang et al. [7]	Q370 + S31603; Q235 + S31603	Welded box-section beam-column	Full-scale test + Numerical analysis	Constant axial load + cyclic lateral load
Gao et al. [10]	Q235 + 304L	X-type gusset plate-to-CHS connection	Test + design-code assessment	Axial tension/compression
Guo et al. [145]	Q355 + 304	Double-shear bolted connection	Test + Numerical analysis + design-code assessment	Monotonic tension
Yang et al. [146]	Q355B + S31603; Q500MD + S31603	Welded tubular T-joint	Full-scale test + design-code assessment	Monotonic brace in-plane bending
Yang and Ban [147]			Numerical analysis + parametric study + design model	Monotonic brace in-plane bending
Yang et al. [148]			Test + Numerical analysis + restoring-force mode	Cyclic brace in-plane bending
Yang et al. [149]	Q235 + 304L	CHS T- and K-joints	Test + design-code assessment	Axial loading

Research on stainless-clad bimetallic steel T-joints has mainly focused on tubular members under monotonic and cyclic loading. Under monotonic in-plane bending, box-section T-joints generally fail by chord plastification, while larger brace-to-chord width ratios may induce combined chord face and side-wall failure [146]. Numerical studies further showed that joint resistance is governed mainly by the brace-to-chord width ratio, whereas the effect of chord length is limited [147]. Under cyclic in-plane bending, the dominant failure mode shifts to fracture of the connecting weld, and the moment resistance decreases relative to the monotonic case, although good ductility and energy dissipation are still observed [148]. For CHS T-joints under axial loading, the observed failure modes and normalized strengths are broadly comparable to those of conventional carbon-steel tubular joints, indicating that existing design approaches may be extended to this material system, albeit with generally conservative predictions [149].

### 4.5. Composite Members

Concrete-filled bimetallic tubular columns are the main form of composite bimetallic members studied so far. Early studies mainly examined circular stainless steel-carbon steel double-layer tubes with concrete infill, and showed favorable composite action, compressive resistance, deformation capacity, and hysteretic performance [150,151,152]. More recent studies shifted to metallurgically bonded bimetallic steel systems. For stainless-clad [153,154] and titanium-clad [155,156] stub columns, tests and numerical analyses confirmed stable interaction between the clad tube and concrete core. The results indicate that concrete strength, substrate steel strength, and section slenderness significantly influenced the compressive response, whereas the effect of clad ratio was generally limited [153,154,155,156]. A further study also extended the concept to concrete-filled double-skin tubular stub columns with stainless-clad outer tubes, suggesting the feasibility of more material-efficient composite configurations [157].

Beyond stub columns, further work has addressed slender columns and section slenderness. Numerical results showed that increasing the length-to-diameter ratio markedly reduced the axial capacity because of flexural buckling effects, and a modified design approach was proposed for such members [158]. At the cross-sectional level, increasing the clad ratio reduced the axial slenderness limit of square concrete-filled stainless-clad bimetallic steel stub columns [12]. This indicates that conventional section classification rules should be further verified for clad composite members. As for the composite walls, Guo et al. [159] showed that the axial compressive behavior of stainless-clad bimetallic steel-plate composite walls was mainly governed by the normalized faceplate slenderness ratio, which controlled local buckling mode and compressive resistance. The subsequent work further demonstrated that replacing conventional concrete with UHPC (Ultra-High Performance Concrete) significantly improved the strength and stiffness of the wall, although at the expense of reduced ductility [160]. For composite tubular joints, T-joints with concrete-filled bimetallic tube chords were investigated under axial tension and compression. The results showed that concrete infill fundamentally changed the failure mode from chord-face plastification to web-member-controlled failure, and markedly enhanced the joint resistance [161].

Overall, the component-level studies in Section 4 suggest that the governing failure mode of bimetallic steel members depends strongly on product form, loading mode, and fabrication-induced material heterogeneity. Thin-walled columns are mainly governed by local or overall buckling, whereas plate girders are controlled by patch loading, shear buckling, web-opening effects, and tension-field development. Joints and connections are more sensitive to weld details, local plasticity, bolt-hole weakening, and fatigue cracking. Concrete-filled bimetallic tubular members benefit from composite action between the clad tube and concrete core, but their resistance is still affected by section slenderness, interface integrity, and confinement efficiency. Existing design methods are mostly extended from carbon steel or stainless steel specifications. This approach is convenient, but it is not fully validated for bimetallic systems because most current design rules are based on homogeneous material assumptions. In bimetallic members, the composite-section stiffness, clad ratio effect, residual stress distribution, and interface-sensitive damage may differ from those of conventional steel members. Therefore, current buckling curves, effective-width methods, section classification rules, and interaction equations should be regarded as preliminary design bases rather than fully validated provisions. Future design models should explicitly consider fabrication-induced material heterogeneity, interfacial bonding quality, residual stress distribution, clad ratio, and service-dependent degradation.

## 5. Conclusions

Bimetallic clad steels provide an effective route to combine the load-bearing capacity and cost efficiency of carbon steel with the corrosion resistance, heat resistance, or special functions of cladding metals. The conclusions of this paper could be summarized as follows:

1. Existing studies show that explosive welding, roll bonding, diffusion bonding, surfacing, and related processes can achieve metallurgical bonding. However, interfacial quality remains sensitive to processing parameters, microstructural evolution, residual stress, and brittle intermetallic phases. Different fabrication routes have different advantages. Explosive welding is suitable for dissimilar clad plates, roll bonding is useful for large-scale plate production, and additive manufacturing offers flexibility for graded or complex components but still needs further development for structural application.
2. The mechanical and durability performance of bimetallic steels is governed by the coordinated response of the substrate, cladding layer, and bonding interface. Current research has clarified their basic tensile, compressive, fracture, fatigue, corrosion, and high-temperature behaviours. In general, the substrate mainly provides strength, while the cladding layer improves durability. Nevertheless, material mismatch may cause local stress concentration, interfacial damage, and premature cracking, especially under cyclic loading, fire exposure, corrosion, or coupled actions. Direct quantitative comparison remains limited by differences in material systems, clad ratios, fabrication routes, specimen geometries, and test conditions.
3. At the structural level, studies have extended from material tests to welded joints, thin-walled members, plates, girders, connections, and composite members. These results confirm the engineering potential of bimetallic steels in durable and sustainable structures. However, existing work is still fragmented. Current design methods adapted from carbon steel or stainless steel specifications provide a useful starting point, but their applicability to bimetallic members still requires further validation, especially when residual stress, clad ratio, local buckling, web openings, and interface behaviour are involved. Unified constitutive models, design methods, and durability evaluation frameworks remain insufficient.

Future research should therefore focus on the following aspects: (i) establishing quantitative process–interface–property relationships for different fabrication routes and material systems; (ii) developing constitutive, fatigue, fracture, and corrosion-degradation models that explicitly consider clad ratio, interface quality, residual stress, and material heterogeneity; (iii) clarifying long-term durability and coupled corrosion–fatigue–fire degradation mechanisms; (iv) evaluating the applicability and safety margin of current steel design provisions for bimetallic members; and (v) developing standardized testing, manufacturing quality-control, inspection, and design procedures. Life-cycle assessment should also be strengthened to support wider engineering application.

## Figures and Tables

**Table 1:** Summary of representative studies on the mechanical properties of bimetallic steels.

References	Bimetallic Steel Grade	Fabrication Route	Loading Condition
Tang et al. [68]	Q235B + S34553	Explosive bonded	Monotonic tensile
Liu et al. [69]	Q235 + TA2	Hot-rolled bonded	Tensile and bend coupon test
Motarjemi et al. [70]	ASTM A516 + A240		Round tensile test; micro-flat tensile test
Dhib et al. [71]	ASTM A283 + A240		Tensile and shear test; Charpy impact test
Liu et al. [72]	Q235 + SUS304	Hot-rolled bonded	Tensile test and fracture characterization
Hu et al. [73]		Not reported	Dynamic tensile test at different strain rates
Yang et al. [75]		Hot-rolled bonded	Quasi-static and dynamic uniaxial tensile tests
Mei and Ban [74]	Q235 + 316L	Hot-rolled bonded	Quasi-static and high-strain-rate compression tests
Yuan et al. [67]	Q355B + 316L	Laser 3D printing	Uniaxial tensile test; Bending test
Kang et al. [11]	Q235/Q355/Q550 + 316L	Laser cladding	Uniaxial tensile test

## Data Availability

Data sharing is not applicable.
